
JOURNAL INFORMATION
==============================
NLM Title Abbreviation: Gastroenterol Rep (Oxf)
Journal ID: Gastroenterol Rep (Oxf)
NLM Journal ID: 101620508
Journal ID: gastro

Gastroenterology Report

EISSN: 2052-0034
Publisher: Oxford University Press

ARTICLE INFORMATION
==============================
PMCID: PMC5421497
DOI: 10.1093/gastro/gox019
Article ID: gox019
Article version: 1
Subjects: Chinese Abstracts

Chinese Abstracts – Volume 5 Issue 2*

* This article abstract was originally published in English. This translation has not been verified and should not be relied upon—it is provided for reference purposes only. The Publishers have not checked this translation and accept no liability for completeness or accuracy of this translation or the use of this translation for whatever purpose. This translation may be incomplete and inaccurate in whole or in part. If you need to rely upon a translation of this abstract, a professional human translator should be engaged to supply an accurate translation of the original English. When referencing articles from this journal, please always refer to the original English version, rather than a translated equivalent.

Print publication date: 2017 May
Electronic publication date: 2017 May 8
Volume: 5
Issue: 2
First page: C1
Last page: C2
Copyright: © The Author(s) 2017. Published by Oxford University Press and Sixth Affiliated Hospital of Sun Yat-Sen University
Copyright year: 2017
License: This is an Open Access article distributed under the terms of the Creative Commons Attribution License (http://creativecommons.org/licenses/by/4.0/), which permits unrestricted reuse, distribution, and reproduction in any medium, provided the original work is properly cited.
License URL: https://creativecommons.org/licenses/by/4.0/

Page count: 2

==============================
OXFORD

*Gastroenterology Report*, 2017, 1–2

doi: 10.1093/gastro/gox019

ABSTRACTS

### Chinese Abstracts – Volume 5 Issue 2*

### Review（综述）

### Diagnosis of cirrhosis and portal hypertension: imaging, non-invasive markers of fibrosis and liver biopsy
### 肝硬化和门脉高压的诊断：影像学、非侵入性纤维化标志物和肝脏活检

[摘要] 肝硬化的概念一直在不断演进, 现在已经明确的是, 代偿期与失代偿期肝硬化预后是完全不同的。进展期慢性肝病（ACLD）这一称谓能更好地反应肝脏组织学变化的进程, 比如, 即使肝硬化形成了, 肝脏组织学仍会继续进展；而在去除病因后, 肝脏也可能会在组织学上逆向转好。对于代偿期ACLD, 门脉高压标志着疾病进展到了一个并发症高危期, 需要准确评估和治疗。在一些复杂的临床情况下, 用以诊断肝硬化（肝脏活检）和门脉高压（肝静脉压力梯度检测）等侵入性检查仍然是至关重要的, 但由于各种非侵入性检查方法在常规病例中的广泛应用, 侵入性检查的临床需求已大大降低。本文对纤维化标志物、弹性成像和影像学等非侵入性检查方法的应用情况、主要优缺点进行了综述。

关键词：代偿性进展期慢性肝病；肝静脉压力梯度；弹性成像；超声

### Novel treatment options for portal hypertension
### 门脉高压症新的治疗方法

[摘要] 门脉高压症多见于肝硬化患者, 是静脉曲张出血、腹水、肝性脑病等并发症的主要推动因素。门脉高压症本身可以作为肝脏失代偿的先兆, 显著影响肝硬化患者的预后。目前, 非选择性β受体阻滞剂（NSBBs）、血管加压素类似物、生长抑素类似物是主要治疗药物, 但这些治疗方案仅仅是针对内脏器官的充血, 其疗效远无法令人满意。而旨在减少肝硬化患者肝内血流阻力的安全有效的治疗方案仍不明确。近年来, 一些体外研究、动物实验及临床试验聚焦于减少肝内血流阻力及其他治疗途径。本文中, 我们重点探讨了这些新的治疗策略的安全性和有效性, 并展望了一些具有潜在应用前景的治疗方法。

关键词：门脉高压；肝静脉压力梯度；非选择性β受体阻滞剂；一氧化氮；血管生成；他汀类药物；法尼醇受体；肾素-血管紧张素-醛固酮系统

### Ascites, refractory ascites, and hyponatremia in cirrhosis
### 肝硬化中的腹水、顽固性腹水和低钠血症

[摘要] 腹水是肝硬化最常见的并发症, 会增加肝硬化患者的并发症发生率和病死率。腹水的形成是由于临床显性门脉高压症引起内脏动脉血管舒张不断加剧、继而导致全身有效动脉血容量的代偿机制丧失。为了维持有效动脉血容量, 血管收缩和抗利钠系统被激活, 以增加血钠浓度, 促进液体潴留。随着内脏动脉血管进行性舒张, 肠道毛细血管压力增高, 加之肝窦高压所继发的毛细血管渗透压增高, 最终导致腹腔内低蛋白液形成。在部分患者中, 液体能通过膈膜窗进入胸腔从而形成胸水。此外也会出现一些感染性并发症, 如自发性细菌性腹膜炎。最终, 随着肝硬化的进展、门脉压力的不断增高、心输出量代偿功能的丧失及内脏血管舒张的进一步恶化, 肾功能也会因肾脏血管收缩、自由水排泄受损及严重的钠潴留而受损, 从而出现顽固性腹水、肝肾综合征和低钠血症等并发症, 这些并发症都会增加短期病死率。目前, 肝移植是惟一的根治方法, 但最近的研究结果也使我们看到了一些替代治疗方案的曙光。本文将对腹水、顽固性腹水和低钠血症的病理生理及治疗策略进行深入探讨。

关键词：腹水；门脉高压症；肝硬化；低钠血症

### Recent advances in the management of variceal bleeding
### 静脉曲张出血的最新治疗进展

[摘要] 急性胃食管静脉曲张破裂出血是肝硬化门脉高压患者最严重的并发症, 常需急诊处理, 有较高的并发症发生率和病死率。即便在最初的急性出血中幸存, 这些患者再次出血及出现其他失代偿事件的风险仍然很高。过去30年治疗策略的不断演进, 已大大提高了静脉曲张出血患者的生存概率。由于肝硬化具有多个病期, 所以单一笼统的治疗模式已被摒弃, 取而代之的是针对不同病期的患者采用不同的治疗方法以得到最佳治疗效果。这需要我们按疾病分期对肝硬化患者进行分层, 然后方能针对个体化病例采用恰当的治疗方式并选择合理的治疗时机。与此同时, 新的治疗方法不断涌现, 而大量最新的证据仍然支持内镜和药物治疗的应用。本文中, 我们对各个病期及止血失败后的治疗选择进行了总结。我们进行了细致的文献综述, 呈现了所有的随机对照试验和meta分析结果, 这些结果支持上述的治疗模式。

关键词：静脉曲张；急性静脉曲张出血；肝硬化；预防；非选择性β受体阻滞剂；静脉曲张结扎术

---

*This article abstract was originally published in English. This translation has not been verified and should not be relied upon—it is provided for reference purposes only. The Publishers have not checked this translation and accept no liability for completeness or accuracy of this translation or the use of this translation for whatever purpose. This translation may be incomplete and inaccurate in whole or in part. If you need to rely upon a translation of this abstract, a professional human translator should be engaged to supply an accurate translation of the original English. When referencing articles from this journal, please always refer to the original English version, rather than a translated equivalent.
本文摘要最初是以英文发表。中文摘译未获认证，谨供参考。出版社没有对中文摘译进行核验，不负责译文的完整性和准确性，也不会将其用以其他目的。中文摘译可能会出现部分不完整或不准确的情况。如果你需要准确获悉摘要，最好能请一位专业译者针对英文原文进行翻译。阅读杂志文章，请参考英文原文，不推荐中文翻译。

### Acute kidney Injury and the hepatorenal syndrome: a review on renal dysfunction in cirrhosis

### 急性肾损伤和肝肾综合征：肝硬化中肾功能异常的综述

[摘要] 肾功能异常是肝硬化的常见并发症，对临床表现和预后有着极为重要的影响。肝硬化患者易出现急性肾损伤（AKI）；肝肾综合征（HRS）则是肾功能异常的一种特殊类型。肾前性AKI、肝肾综合征型AKI（HRS-AKI，以往称为1型）和急性肾小管坏死（ATN）是肝硬化中AKI最常见的类型。由于治疗策略不同，准确区分这些AKI类型是必要的。血浆容量扩张治疗对肾前性AKI有较好的效果，但HRS-AKI和ATN病死率较高，需要不同的治疗措施。最近，一些诊断标准（如以2.5 mg/dL为界值的血肌酐水平诊断HRS-AKI）已被摒弃，用以HRS-AKI及其他类型的AKI进行早期诊断和治疗的新的尿液标志物则成为了研究热点。本文从病理生理、诊断、治疗策略方面对HRS-AKI与其他类型AKI的临床差异进行了全面探讨。

关键词：肝硬化；急性肾损伤；肝肾综合征

### Hepatic encephalopathy

### 肝性脑病

[摘要] 肝性脑病（HE）是发生于进展期肝病患者的一种可逆的大脑功能损伤综合征。HE确切的病理生理机制尚未存争议，相关假说主要聚焦于神经毒素的作用、由于肝衰竭代谢改变引起的神经传导功能受损、大脑能量代谢的变化、系统性炎性反应及血脑屏障的改变。HE可产生各种各样的神经心理学表现。轻微型HE主要是通过心理测试异常来进行诊断。临床显性HE包括性格变化、进行性时空意识障碍、嗜睡、昏迷，除了临床研究外，无需任何特殊的心理测试来协助诊断。HE可基于基础疾病、临床表现的严重程度、病程长短及存在的诱因进行分类和分级。临床显性HE的治疗包括支持治疗、诱因治疗、乳果糖和/或利福昔明。轻微型HE的常规治疗仅推荐用于部分选择病例。

关键词：肝性脑病；病理生理；诊断试验；治疗策略

### Portal vein thrombosis in patients with cirrhosis

### 肝硬化患者门静脉血栓形成

[摘要] 门静脉血栓形成（PVT）在肝硬化患者中较为常见，尽管PVT相关的严重并发症（如肠系膜缺血）很罕见，但一旦出现足以危胁生命。目前PVT的临床意义、诊断和治疗仍未明确，尚处于探讨中。本文对PVT的分级、临床表现地域差异、诊断标准、病理生理及危险因素进行了阐述，对各种治疗方案和预防措施进行了评价，重点分析了各种方案/措施的缺陷。此外，我们还对PVT对预后的影响这一争议问题进行了探讨，着重考虑了肝移植和未来展望。

关键词：门静脉血栓；肝硬化；血栓形成试验；低分子量肝素；经颈静脉肝内门体分流术；肝移植

### Case Report（病例报告）

### Subtle presentation of active primary biliary cirrhosis in chronic hepatitis B: A case report

### 慢性乙型肝炎患者出现原发性胆汁性肝硬化不易察觉的表现：一例报告

### Progressive liver failure post acute hepatitis A, over three month period, resulting in hepatorenal syndrome and death

### 急性甲型肝炎后进行性肝衰竭：三个月期间出现肝肾综合征并死亡
